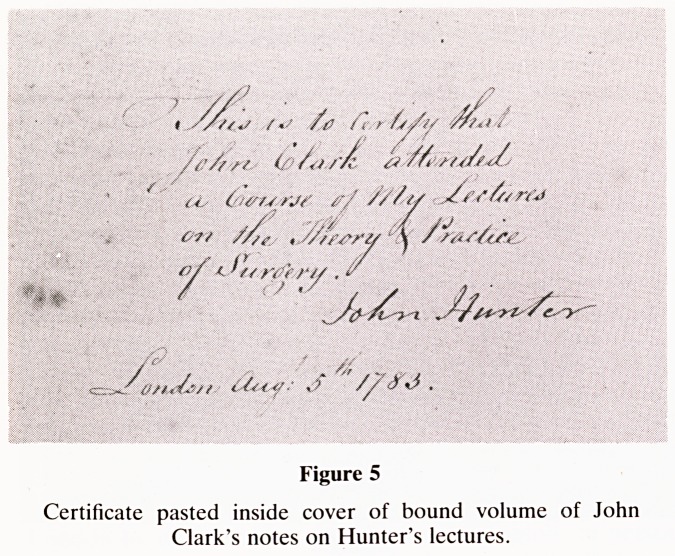# Some Plymouth Worthies, Part 1

**Published:** 1990-03

**Authors:** Michael Reilly

**Affiliations:** Emeritus Consultant Surgeon, Plymouth


### Some Plymouth Worthies (Part 1)

**Michael Reilly** M.S. F.R.C.S.
Emeritus Consultant Surgeon, Plymouth

Most towns in this country have produced distinguished sons who have made valuable contributions to art and science in their time. Plymouth is no exception. This paper deals with some names, well-known or less recognised, who practised medicine and surgery in Plymouth during the 17th, 18th and 19th centuries. Nearly all were Plymouth men, a few came from outside and one or two made their names in distant parts.

Those who feel confined by the modern tendency to believe that original ideas can only fructuate in 'centres of excellence' as the result of controlled excercises in research projects, and that success can only be achieved by climbing prescribed ladders, may take heart from the story of some past 'Plymouth Worthies' who overcame the obstacles that they met in their time. There is still a place for individual initiative in an increasingly regimented society.

In the 17th century the wool trade made Devon, after Yorkshire, 'the most populous and industrious county in the whole of England', in the words of Daniel Defoe. We would now say 'industrialised'. Plymouth was a thriving town, which owed much to Sir Francis Drake. It was he who was responsible for the first reliable water supply from Dartmoor to Plymouth by the construction of Drake's Leat. Parts of this still exist. This feat of engineering is commemorated at the annual Fyshinge Feast, held at Burrator Reservoir, where the celebrations begin with a ceremonial toast to Drake's memory—drunk in water—before proceeding to stronger beverages.

In Plymouth, on February 27, 1647, Joanna Yonge presented her surgeon husband, John, with the second of three sons: James (figure 1). (The Dictionary of National Biography erroneously gives the date as May 11, 1646). James was a bright boy, already able to read and write well, when he was sent ot Mr. Horsman's Latin School at the age of nine. He only remained there for two years because his father, 'a victim of a lingering disease', wished to see his sons launched before he died. For some reason his father seemed to favour the other brothers and to have neglected James. The elder brother, John, was apprenticed to Thomas Spenser, Surgeon of the Hospital of Plymouth. James was apprenticed for eight years to Silvester Richmond, of Liverpool, a surgeon to the ship of war 'Constant Warwick'. So, at the age of eleven, began a medical career of which the first fourteen years were spent mainly at sea. There he probably learned to appreciate the force of Drake's earlier dictum: 'I must have the gentleman to haul and draw with the mariner and the mariner with the gentleman. I would know him that would refuse to set his hand to a rope, but I know that there is not any such here.' Mutatis mutandis, physicians then were very much the gentlemen and surgeons the mariners, but they have since drawn somewhat closer together.

During his first experience at sea Yonge visited Portugal and North Africa, where he was present at the ineffectual bombardment of Algiers in 1662. On returning to England he was paid off and worked for four months as an apothecary's assistant in Wapping. He then assisted in his father's practice for six months, but complained of being kept short of money and clothes, even shoes when they were worn out. He was happy to return to sea again, this time on a voyage of inspection to the cod fisheries in Newfoundland. In 1664 he visited West Africa and the Mediterranean. In the Lipari islands he was asked to tap a brother of the Governor for ascites. Shrewdly he diagnosed that the patient had been bled too much by a priest acting as a physician. The priest was obviously in breach of the edict of the Fourth Lateran Council (A.D. 1215) which prohibited all persons in Holy Orders from letting blood: 'Ecclesia abhorret a sanguine'. Yonge refused to prescribe anything 'but some gentle things, which gave him some ease'. He records in his journal 'for this service I had but poor reward, except good entertainment, guns and much compliment'.

A more curious case which he encountered on the same island was one of true hermaphroditism, accidentally revealed, which he investigated in the meticulous manner which was to distinguish his later work. He records 'I made divers enquiries, such as my abilities then put me upon, and looking over my books found the like *had* happened in the world, all of which are contained in the history of this accident, as I have written it in the first volume of my observations'.

In 1665 Yonge's ship returned to Plymouth, where he found that it was the day of his sister's wedding. He obtained leave of absence, which was fortunate for him as the ship returned to London at the beginning of the Plague. After later rejoining his ship he was captured by the Dutch and spent eight months in Amsterdam as a prisoner of war. He escaped typhus by removal to hospital, but an attempt to escape himself was unsuccessful. He was finally released on an exhange of prisoners in time to see the aftermath of the Great Fire of London in 1666: 'divers heaps of rubble yet smoking'.

**Figure 1**

James Yonge (1647–1721). From a portrait in the possession of Commander P. Yonge, RN.

**The material for this paper was collected for the 1985 Bradshaw Lecture, given at the Royal College of Surgeons of England, in Lincoln's Inn Fields. The lecture was composed mainly of commentary on some forty slides, and has therefore not been published. Thanks are given to the College for permission to publish this version.**

He returned to Plymouth and spent two years there 'in study, practice and sometimes riding in the country'. In 1668 he made his final voyage to Newfoundland, after which—at the age of 22—he produced a valuable and detailed report on the methods and management of the fisheries there. He then settled permanently in practice in Plymouth, where he was appointed surgeon to the Naval Hospital—in fact a ship—at the rate of five shillings a day: 25 modern pence. In 1674 he was appointed Deputy Surgeon General to the Navy.

In 1678 Yonge visited London for a medical symposium, no doubt accompanied in the ancient Greek manner by liquid refreshment. As a result he published his well-known 'Currus Triumphalis a Terebintho' (figure 2) on the treatment of haemorrhage with turpentine, but containing too some valuable general observations. He kept up correspondence with many notable names in medicine and science and also read widely in other fields, including Cervantes and Rabelais.

He became Mayor of Plymouth in 1694 and in 1702 was examined for the licence of the Royal College of Physicians of London by its President, Sir Thomas Millington, in person. He had previously practised on the licence of the Bishop of Exeter. He passed the examination and was admitted as a Licentiate. He was then aged 55. The same year he was elected Fellow of the Royal Society.

During his practice in Plymouth Yonge published numerous medical and surgical papers. He was the first surgeon since Heliodorus, in the first century A.D., to describe in detail the use of a flap to cover an amputation stump, instead of carrying out the old circular procedure. He succeeded probably because he was neat and meticulous. He was generous enough to give credit for the idea to Mr. C. Lowdham, a surgeon's assistant in Exeter. Yonge describes division of both bones in amputations below the knee or elbow, scaling the bones and greasing the ends. It was not until the time of Lister, two hundred years later, that flaps could be used routinely without the fear of sepsis.

One of Yonge's best known publications is 'Wounds of the Brain proved Curable' (figure 3), the happy result of a difference of opinion with a Plymouth physician, Dr. Durston. Yonge had recounted to a local medical audience the case of a four year old boy, injured after swinging on a gate. The gate had come off its hinges and crushed the boy's head between a boss near the latch and a stone on the ground. Yonge removed a piece of bone from the wound caused by the boss, and treated it with débridement and dressings.

108 *Currus Triumphalis,*

*A new Way of Amputating large Members, and a more ſpeedy convenient Method of curing Stumps, than that commonly practiſed.*

*Diſcovered in a Letter, to his eſteemed Friend, Mr.* THO. HOBS, *Chirurgeon in* London.

*SIR,*

I Find by yours, that you are ſurprized with the intimation I gave you, of a way of amputating large Members, ſo as to be able to cure them *per Symphyſin*, in three weeks, and without fouling, or ſcaling the bone. It is a Paradox that I will now evince to you to be a truth, after I have firſt taken notice of what you affirm, that there is a neceſſity of ſcaling the ends of thoſe bones, left bare after the uſual way of diſmembring, before the Stump can be ſoundly cured; that you never yet found it otherwiſe but that where it

**Figure 2**

Reproduction of p.108 of Yonge's 'Currus Triumphalis', in which he describes his successful method of dealing with amputation stumps.

WOUNDS OF THE BRAIN Proved CURABLE,

Not only by the Opinion and Experience of many (the beſt) Authors, but the remarkable Hiſtory of a Child four Years old cured of two very large Depreſſions, with the loſs of a great part of the Skull, a Portion of the BRAIN alſo iſſuing thorough a penetrating Wound of the *Dura* and *Pia Mater.*

Publiſhed for the Encouragement of Young Chirurgeons, and Vindication of the Author,

*JAMES YONGE.*

*Hæc dixi ut contradicerem* Opinioſis, *qui non credunt cerebrum poſſe ſanari,* ——*quia ego cognoſco,* & Cerebrum *ſanari, & alias medullas.* Jac. de Carpio, Tract. de Fr. Cranii.

*LONDON,*

Printed by *J. M.* for *Henry Faithorn* and *John Kerſey,* at the *Roſe* in S^t *Pauls Church-yard.* 1682.

**Figure 3**

Reproduction of title page of 'Wounds of the Brain proved Curable'.

There was a smaller depressed fracture on the other side which he was unable to elevate. The boy made a good recovery.

Dr. Durston, possibly moved by professional jealousy, affirmed that all authorities, including the celebrated M. Defoy and M. Runevief, agreed that wounds of the brain were invariably fatal. There must therefore have been some deception. A splendidly acrimonious argument followed, with charges of fraud and counter-charges of blind bigotry. Durston was unwise enough to say that Yonge was one of a company of ignoramuses fit for nothing but to cut corns. Yonge compared Durston's intelligence with that of a sheep from which the brain had been removed. It is sad that the laws of libel prevent us from enjoying such an exchange today.

Yonge set to work to vindicate his views. His book starts, in the manner of the time, with a flattering dedication to Sir Hugh Piper, Lieut. Governor of The Citadel in Plymouth, recounting the affair and enlisting his support as a referee: 'One who hath more bravely vindicated his honour by his sword than ever any writer did an hypothesis by that sharper weapon, his pen.' He goes on to describe the case in detail, with line drawings of the gate and its knobs and of the piece of bone removed. He cites in support 64 authors, from Galen and Fallopius to Ambroise Paré, who had said that wounds of the brain were *not* absolutely mortal. He gives a commentary and draws five main conclusions, the last of which is 'that the dura mater doth not always adhere to the skull but trepanning is safe and necessary and that M. Defoy and M. Runevief are in error.' Yonge had clearly demonstrated the occurrence of extra-dural haemorrhage, hitherto believed to be impossible. The book ends with a withering and comprehensive commination of Dr. Durston, who was never heard of again.

In 1679, the year of his success with the depressed fracture. Yonge was reconciled with his father. On his death-bed, twenty-one years after he first thought that he was dying, the old man asked for James' forgiveness and gave him a much-appreciated £50. The book on Wounds of the Brain was not published till three years later.

James Yonge died in 1721 and is buried in St. Andrew's, the City Church of Plymouth.

In the 18th century one of the most noteworthy happenings in Plymouth was the building of the new Royal Naval Hospital at Stonehouse (figure 4). In 1744 the Navy Board presented a Memorial to His Majesty in Council, proposing to build hospitals in Portsmouth, Plymouth and Chatham. The foundations of the hospital at Haslar, in Portsmouth, were laid in 1746 and it was receiving patients by 1753. In Plymouth there were no hospitals for the sick or wounded, only the hospital ship 'Canterbury'. The delay in building a hospital in Plymouth was deliberate: the authorities wished to learn from the experience at Haslar first, so that any mistakes could be rectified in later buildings. The result was a hospital of novel design, widely regarded as the finest in Europe, and far in advance of its time. In 1756 a London architect, Alexander Rovehead, was appointed as 'overseer, and to be in constant attendance at the works'. It is believed that Rovehead worked from plans by William Robinson, whose work in London showed a remarkably similar style. The Seven Years' War had begun in 1756 and must have stimulated the speed with which the hospital was constructed, although it was not completed until 1762. It was built on the Block System, designed to isolate infection, and is the earliest example in this country. It was visited in 1787—a time of peace—by two prominent French scientists, Jacques Tenon and Charles-Augustin Coulomb. They had already visited most of the hospitals in Europe and gave it precendence over all others in regard to 'the judicious construction and distribution of the buildings'. The interesting fact emerges that the Royal Naval Hospital became the pivotal institution for the reform proposals of the French *before* the Revolution, and

**Figure 4**

Copy of a water colour of the Royal Naval Hospital, Stonehouse, Plymouth, in the possession of the Hospital.

for the construction of 'pavilion' or 'block' type hospitals even a century later.

After the death of James Yonge no naval surgeons of sufficient calibre seem to have been available to staff the new hospital. Two civilian surgeons were appointed: Mr. Geach and Samuel Fuge. Fuge was a founder member of the Plymouth Medical Society, described later. Geach was noted as a 'man of superior learning and discernment'. It is related that he was kind and humane, and earned the gratitude of both service and civilian patients. Late one night he was alone visiting a patient on the outskirts of the town, where frequent robberies and a murder had been committed, when he was stopped by two men and nearly dragged off his horse. On seeing his face one man exclaimed 'It is Dr. Geach!', and they both left him and ran off. Modern muggers might not be so considerate.

The best known naval man to be appointed Surgeon during this era was Irish: Sir George Magrath, K.C.B., M.D. of St. Andrew's, F.R.C.P. of London and Edinburgh and Member of the Royal Irish Academy. He was Flag Medical Officer to Nelson, and saw service at Copenhagen and Camperdown. He missed Trafalgar as he was then stationed ashore at Gibraltar. He was Superintendent of a Mediterranean hospital and Inspector of Fleets. When he left the Navy he set up practice in Plymouth, where he became one of the early members of the Plymouth Medical Society. He took an interest in the conditions at Dartmoor, which was built for French prisoners of war and only later became a prison for convicts in 1860. He was somewhat of a dandy, and wore a wig and padded calves long after they had gone out of fashion. He remained a bachelor, but his Irish charm and his interest in the opposite sex provided a fruitful source of scandal until the day of his death. At his funeral in 1857 a disorderly mob is said to have stolen the emblems of his orders, honours and decorations from his coffin.

After the Royal Naval Hospital had been open for a while it was visited by John Wesley, the evangelist, and by John Howard, the prison reformer. They both gave it high praise, but later Thomas Trotter, Physician to the Fleet, found it to be 'a very sink of iniquity and squalor'. The nurses were 'given to drunkenness, debauchery, moral frailty and crime' and the medical staff were accused of lining their pockets from private practice. Nurses have a better press these days, but criticism of doctors dies hard.

A complete overhaul of the administration was recommended, and we have a foretaste of the institution of

This is to Certify that John Clark attended a Course of My Lectures on the Theory & Practice of Surgery.
John Hunter
London Aug: 5th 1783.

**Figure 5**

Certificate pasted inside cover of bound volume of John Clark's notes on Hunter's lectures.

Managers. A remarkable man—a naval officer, be it noted, not an accountant—was appointed Governor. Captain Richard Creyke took up his post, intended to be for three years, in 1795. He remained in charge for 31 years, until his death at the age of 80. He was far-sighted, firm but humane, and by discipline combined with commonsense put the hospital on the road that it has travelled ever since.

There were three other figures, not connected with the Navy, who contributed to the advancement of medical and surgical knowledge in Plymouth during the 18th century. The first is John Huxham, who was born in nearby Totnes in about 1692. He studied medicine in Holland at Leyden, under the famous Boerhaave, and then in France at Rheims, where he took his M.D. He settled in practice in Plymouth. He was 'a classical scholar, well read in works of antiquity, and the author of several works highly esteemed at that time'. He was of sufficient distinction to be elected a Fellow of the Royal Society, but his present interest to us is in connection with a case treated by Dr. Spry. An old man of 94, who was present at the Eddystone Lighthouse fire in 1754, looked up into a shower of molten lead. Some ran down his throat. Dr. Spry treated him for several days until he died, and then performed an autopsy. He removed from the stomach a lead cast weighing 7oz. 5dr. 18gr. Spry sent a report to the Royal Society, who refused to believe him. Dr. Huxam became interested, and suggested an experiment. John Hunter would have approved this approach! Spry fed molten lead to a fowl and to two dogs, one of which survived. After this his paper was accepted.

Of the other two figures one is unnamed, but is said to be the first to perform a tenotomy for talipes. The last is John Clarke, Junior, who was inspired to visit London to sit at the feet of John Hunter (figure 5). His complete notes of Hunter's lectures, with other valuable books and documents, were in the archives of the Plymouth Medical Society. They had all been removed and dumped in the cellars of a peripheral hospital to make way for administrative expansion. They remained lost for many years until diligence and serendipity resulted in their recovery.

The volume of Clarke's notes provides some of the fullest records that we have of Hunter's lectures, and are the only ones that we have that were dated and signed by John Hunter himself. They can be used to trace changes in Hunter's earlier and later methods and ideas. They are at present on permanent loan to the library of the Royal College of Surgeons of England, where full transcripts have been made.

**Part 2 will appear in the next number.**

## Figures and Tables

**Figure 1 f1:**
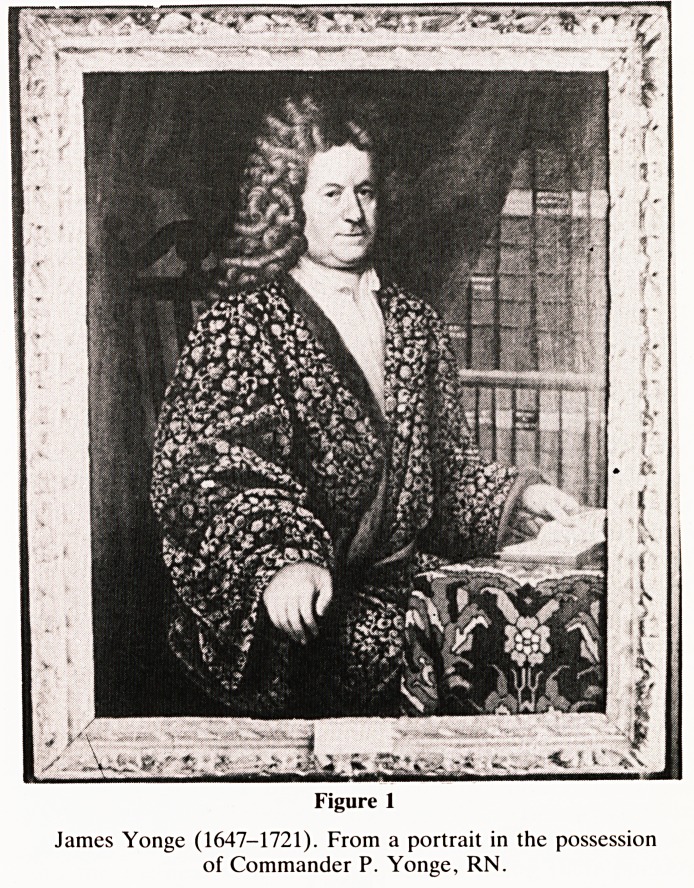


**Figure 2 f2:**
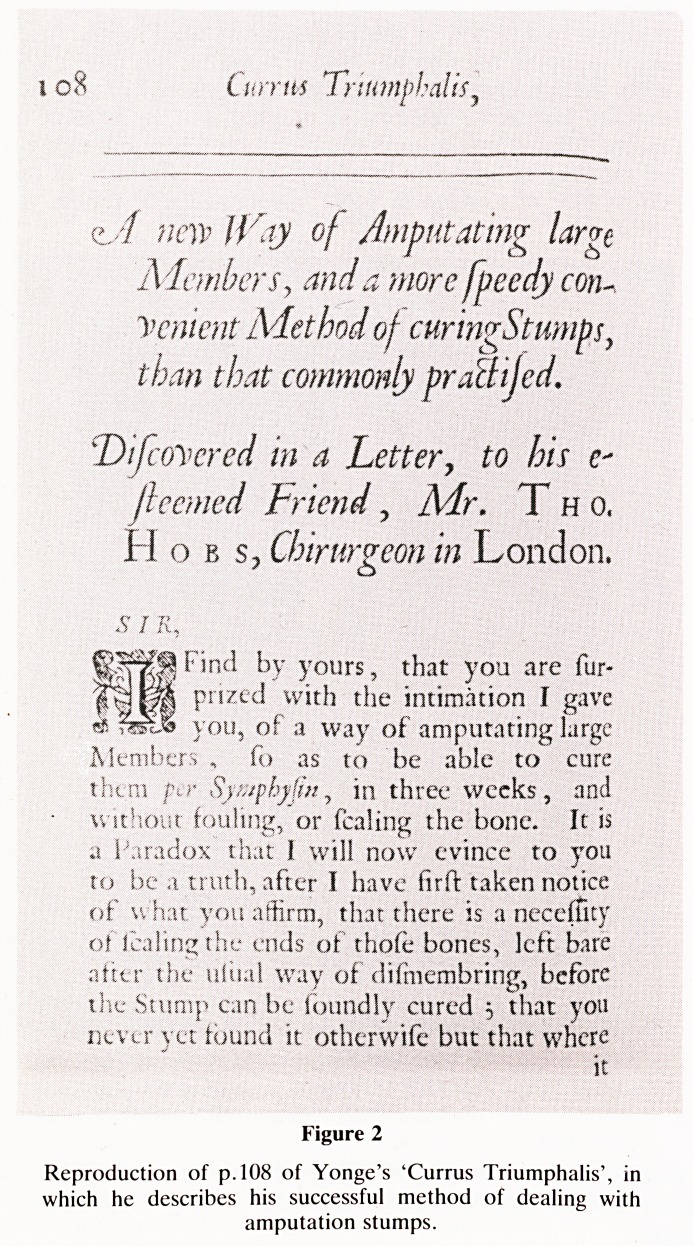


**Figure 3 f3:**
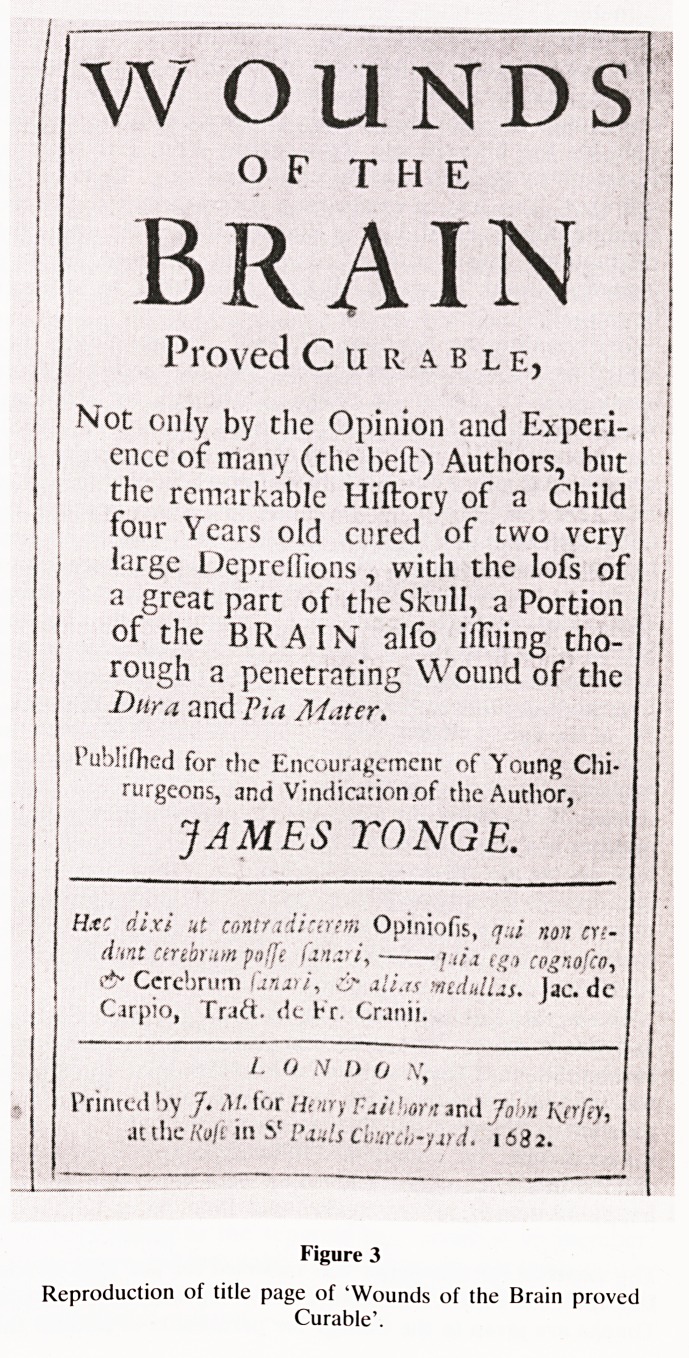


**Figure 4 f4:**
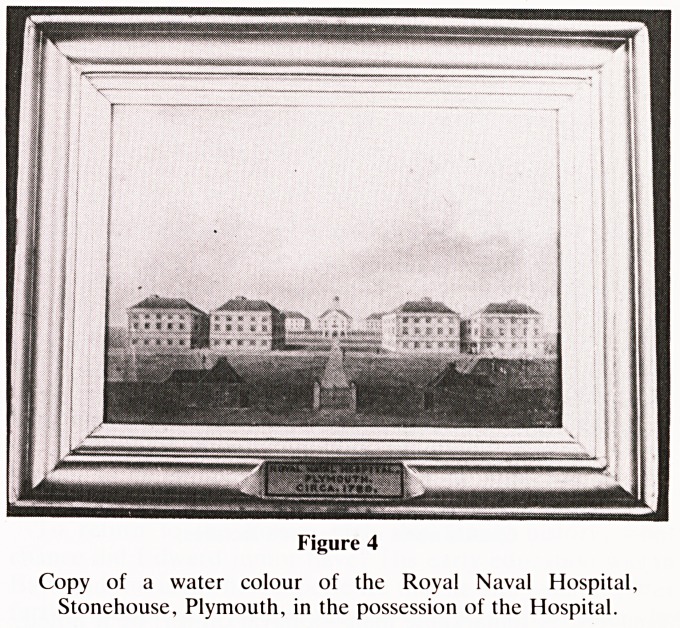


**Figure 5 f5:**